# Air Space Proportion in Pterosaur Limb Bones Using Computed Tomography and Its Implications for Previous Estimates of Pneumaticity

**DOI:** 10.1371/journal.pone.0097159

**Published:** 2014-05-09

**Authors:** Elizabeth G. Martin, Colin Palmer

**Affiliations:** 1 School of Ocean and Earth Sciences, University of Southampton, Waterfront Campus, Southampton, United Kingdom; 2 School of Earth Sciences, University of Bristol, Bristol, United Kingdom; College of the Holy Cross, United States of America

## Abstract

Air Space Proportion (ASP) is a measure of how much air is present within a bone, which allows for a quantifiable comparison of pneumaticity between specimens and species. Measured from zero to one, higher ASP means more air and less bone. Conventionally, it is estimated from measurements of the internal and external bone diameter, or by analyzing cross-sections. To date, the only pterosaur ASP study has been carried out by visual inspection of sectioned bones within matrix. Here, computed tomography (CT) scans are used to calculate ASP in a small sample of pterosaur wing bones (mainly phalanges) and to assess how the values change throughout the bone. These results show higher ASPs than previous pterosaur pneumaticity studies, and more significantly, higher ASP values in the heads of wing bones than the shaft. This suggests that pneumaticity has been underestimated previously in pterosaurs, birds, and other archosaurs when shaft cross-sections are used to estimate ASP. Furthermore, ASP in pterosaurs is higher than those found in birds and most sauropod dinosaurs, giving them among the highest ASP values of animals studied so far, supporting the view that pterosaurs were some of the most pneumatized animals to have lived. The high degree of pneumaticity found in pterosaurs is proposed to be a response to the wing bone bending stiffness requirements of flight rather than a means to reduce mass, as is often suggested. Mass reduction may be a secondary result of pneumaticity that subsequently aids flight.

## Introduction

Pterosaurs were a group of flying archosaurian reptiles that evolved during the Triassic, making them the earliest vertebrates capable of powered flight by over 50 million years. They also diversified and evolved rapidly and to great sizes, including the largest-ever flying animal, *Quetzalcoatlus northropi,* with a wingspan of over 10 m. Although pterosaur flight capabilities have been greatly debated (e.g. [1]–[5]), numerous physiological adaptations such as a bird-like respiratory system [6], progressive neurological systems [7] and well developed flight membranes [8] suggest these animals were very capable volant organisms.

The bird-like respiratory system present in pterosaurs [6] comprised of lungs and an extensive air sac system with diverticula that pneumatized the postcranial skeleton. Although this is unique to birds among extant animals, it appears to have been common among extinct non-avian archosaurs, including sauropodomorph dinosaurs [9]–[11], non-avian theropod dinosaurs [9], [12], [13], and pterosaurs [6], [9], [14]–[17].

Pneumaticity is most often studied on a presence or absence basis (e.g. [9], [15], [18]), but can also be quantified through a relative pneumaticity index [12], [19], or by looking at the volume of pneumatic cavities. This air space proportion (ASP), is defined as the proportion of bone cross section occupied by air space compared to the total cross-sectional area [10]. Essentially, it gives the percentage of bone area taken up by air. Like pneumaticity index, this value can be used for comparison between specimens and taxa, and with respect to the degree of pneumatization seen throughout an evolutionary stage. ASP is a relatively new way of measuring pneumaticity that has been applied mainly to sauropods (e.g. [20]), but also once to pterosaurs [17]. As ASP is a new metric, it can be difficult to compare these values to comparable characters found in the literature. In terms of long bones, the *K* value has been used to assess the cortical thickness as a ratio of inner to outer bone radius, which allows for comparison between taxa, and has been used in birds [21]–[23] and pterosaurs [24]. Geometrically, ASP is equal to *K* ^2^ for a given circular long bone shaft of constant cortical thickness, which allows the conversion of published *K* values to ASP values.

Previous fossil ASP and *K* studies have one thing in common: they were measured from a single section of the bone that was usually where the specimen has been broken and exposed naturally, which tends to be in the shaft. These studies have not looked at how ASP might vary throughout the bone, and therefore the values used from single shaft cross section are assumed to be representative of the entire bone. The advent of computed tomography (CT) scanning provides a non-destructive way of investigating ASP throughout a bone. While CT studies on pterosaurs in the past have focused on axial [6] and cranial [7] characteristics, more recent studies have focused on the wing bones to answer locomotory questions [25], [26].

Here, previously reported pterosaur wing bone CT scans [25], [26] and additional scans of wing bones (Table 1) were analyzed to calculate ASP throughout individual bones. These values were compared to previously published ASP and *K* values from birds, sauropods, and pterosaurs.

**Table 1:** Details of nine wing bone specimens from numerous pterosaurs where WP indicates wing phalanx.

Specimen	Bone	Formation	Taxonomic Group	Completeness (length)
NHMUK PV R3880	WP?	?Vectis, UK	Istiodactylidae indet.	fragment
NHMUK PV OR35228	WP?	Upper Greensand, UK	Ornithocheiridae indet.	fragments
NHMUK PV OR39411	WP1	Lower Chalk, UK	*Ornithocheirus* sp.	80% complete*
NHMUK PV OR41637	WP1	Lower Chalk, UK	Ornithocheiridae indet.	75% complete*
Uncatalogued UP WP1	WP1	Santana, Brazil	Ornithocheiridae indet.	complete
Uncatalogued UP WP2	WP2	Santana, Brazil	Ornithocheiridae indet.	complete
Uncatalogued UP WP3	WP3	Santana, Brazil	Ornithocheiridae indet.	90% complete*
USNM 11925	Humerus	Upper Chico or Lower Horsetown, USA	*Bennettazhia oregonensis*	complete

*indicates estimate.

Institutional Abbreviations: NHMUK, Natural History Museum, London, UK; SMNK, Staatsliches Museum für Naturkunde Karlsruhe, Karlsruhe, Germany; UP, University of Portsmouth, Portsmouth, UK; USNM, National Museum of Natural History, Washington, D.C., USA.

## Materials and Methods

CT scans from nine pterosaur wing bones from ornithocheirid, istiodactylid, and tapejaroid pterosaurs were analyzed (Table 1). Six specimens are identified as ornithocheirid wing phalanges: three first wing phalanges (WP1), one second wing phalanx (WP2), one third wing phalanx (WP3), and fragments from a wing phalanx of indeterminate position. In addition, an indeterminate istiodactylid wing phalanx and a humerus from the tapejaroid *Bennettazhia* were analyzed. All specimens studied are pterodactyloid pterosaurs. CT scans of *Rhamphorhynchus* yielded no information due to the density of the matrix and bone, while other basal pterosaurs have not yet been scanned as they have not been made available to study. In this paper, the term “end-specimen” refers to a bone shaft with at least one complete proximal or distal end, while the term “fragment” indicates specimens where only a shaft fragment is present.

**Figure 1:**
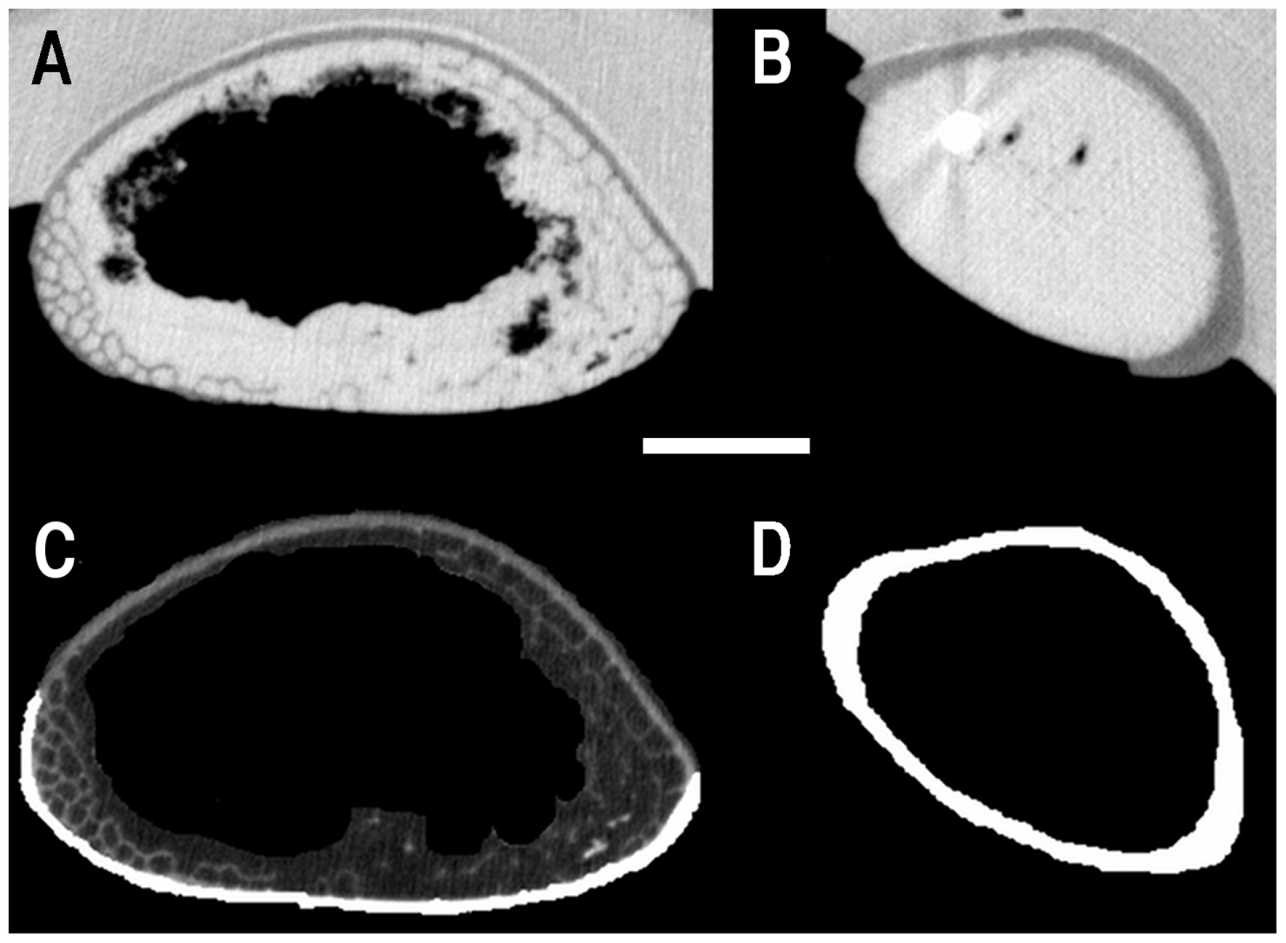
CT scan images from two different regions of pterosaur first wing phalanx. A and B show the unmodified CT scans from A) the distal end of UP WP1 and B) the mid-shaft of UP WP1, while C and D show the modified and corrected images used in the calculation. Air space proportion (ASP) is calculated by determining the cross-sectional area of the internal, air filled cavity (the black centre of D) and dividing that by the total cross-sectional area, including the white cortical tissue and the black cavity. In areas with trabeculae, like C, the calculation of the air space includes the air found in individual trabeculae around the edges. Scale = 10 mm.

**Figure 2:**
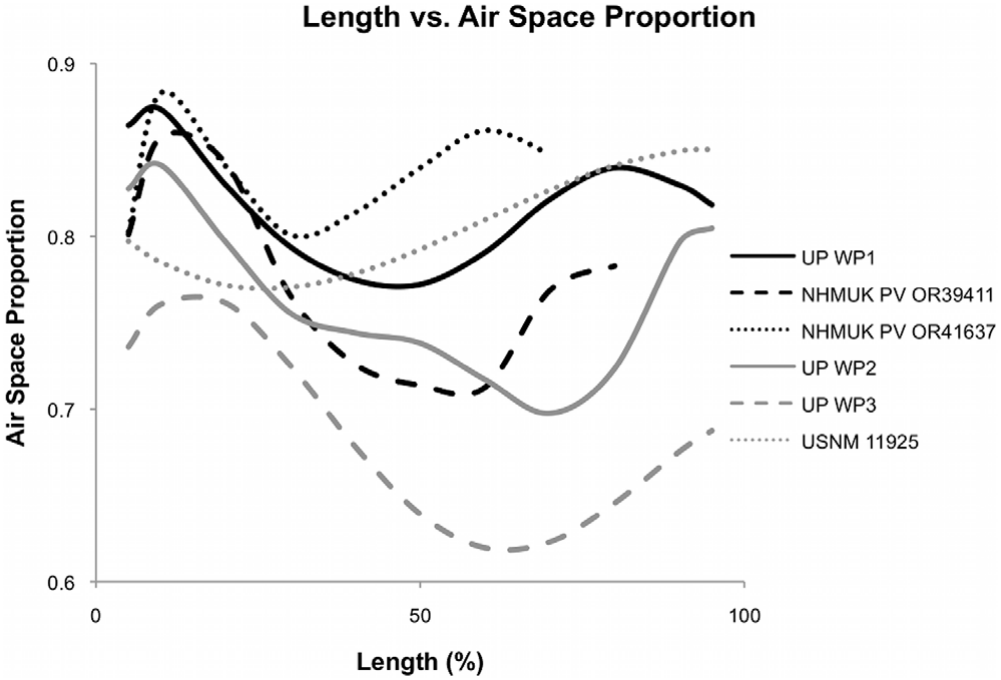
Plot of air space proportion over the length in six pterosaur wing bones. These plots show a polynomial line fit for each bone to show the general shape distribution. Exact measurements can be seen in Table S1.

Cortical area was determined for each scan using the method in Martin and Palmer [26]. Total area for each slice was determined using the same method with the entire filled-in cross-section (Fig. 1). Once cortical area and total area were known, ASP was determined by subtracting the ratio of cortical area to total area from one, which gives the proportion of the total area occupied by air. This was done at set increments throughout the bones for the end-specimens, varying depending on the length of bone and distance between the scans (see Table S1), and two to four times in the fragments depending on the bone length. Plots of length vs. ASP (Fig. 2) show the variation of ASP throughout the bone and values were compared to those found in previous studies of birds, sauropod dinosaurs, and pterosaurs.

## Results

The ASP distributions for each wing phalanx show a similar pattern to the bone volume distribution for the same bones in Martin and Palmer [26], with the ends of the bone having proportionally higher air space than the shaft (Fig. 2). However, ASP shows higher variability than volume within a single bone which is mostly constant throughout the shaft [26]. While WP1 and WP2 appear to be similar in their ASP value and distribution, the single WP3 in our dataset (UP WP3) has a lower central shaft ASP value than any other bone studied, 15% lower than the proximal head, and 10% lower than the lowest ASP recorded in any other bone. However, UP WP3 maintains the pattern of high ASP in the ends and lower in the shaft. While all wing phalanges exhibit the same general pattern, the humerus (USNM 11925) does not, with the ASP generally increasing from the proximal to distal end, and with a marked increase between 55% and 70% of the length of the bone. The fragments were found to have average ASP values lower than end-specimens (Table 2). There is a 5–19% difference between the average ASP calculated and the lowest ASP found in the end-specimens, and a 10–29% difference between the lowest and highest measured ASP values (Table 2).

**Table 2:** Values of Air Space Proportion in nine pterosaur wing bones.

Specimen	Specimen Grade*	Range of ASP	Average ASP
NHMUK PV R3880	fragment	0.71–0.77	0.74
NHMUK PV OR35228	fragment	0.74–0.76	0.76
NHMUK PV OR39411	End-specimen	0.68–0.88	0.77
NHMUK PV OR41637	End-specimen	0.70–0.88	0.83
UP WP1	Complete end-specimen	0.77–0.87	0.81
	(Th 57)^1^	0.56–0.87	0.79
UP WP2	Complete end-specimen	0.69–0.84	0.76
UP WP3	End-specimen	0.59–0.76	0.68
USNM 11925	Complete end-specimen	0.77–0.85	0.81

*Definitions for fragment and end-specimen can be found in text.

1 Indicates specimen that was thresholded at a lower value of 57 to test the affect of thresholding on the results.

## Discussion

### Comparison with Previous Studies

ASP values found in this study are within the range previously published in the literature for pterosaurs (Tables 2, 3), although important differences are discussed below. The ASP values also appear to be within the range of cervical vertebrae of sauropod dinosaurs with high ASP, although they do not exhibit the wide range seen in sauropods [20]. However, these ASP values do exhibit a higher percentage of air in the bones than do bird wing bones [21]–[23] (Table 3).

**Table 3:** ASP values from previous studies.

	Taxonomic Designation	Element (if known)	ASP
**Pterosaurs**	*Pteranodon ingens* [21] *	Humerus	0.90
		Radius	0.81
		Ulna	0.90
		Metacarpal	0.86
		WPs	0.74–0.88
	Dsungaripteroid [24] *	Unknown	0.26–0.28
	Azhdarchoid (juvenile) [17]	Rib	0.77
		WP	0.27
		Femur	0.66
		Tibia	0.72
		Cervical vert.	0.83
**Birds**	Numerous species [21] *	Humerus	0.46–0.74
		Radius	0.28–0.64
		Ulna	0.29–0.64
		Femur	0.38–0.76
		Metatarsal	0.21–0.74
	Several species [22] *	Marrow-filled	0.11–0.69
		Air-filled	0.49–0.71
	Hooded crow and black-billed magpie [23] *	Humerus	0.52–0.67
		Femur	0.55–0.67
		Tibiotarsus	0.34–0.61
**Sauropods**	Numerous species [20]	Cervical vert.	0.28–0.89
		Dorsal vert.	0.36–0.78
		Presacral vert.	0.65–0.85
		Caudal vert.	0.47

*indicates ASP values converted from *K* values.

Vert. is short for vertebra.

Values reported for *Pteranodon* [21] (and derived from previously published literature [1]) are higher than those found here. However, the results of *Pteranodon* ASP may not be accurate because the original cortical thickness values [1] have been transformed first into *K* [21], then additionally into ASP here. It has also been suggested that the initial transformation into *K* was not accurate [21]. Alternatively, as different taxa exhibit contrasting degrees of pneumaticity and wall thicknesses (see below), it may be that *Pteranodon* surpasses other pterosaurian taxa in its proportion of air to bone. The limited sample size and phylogenetic spread of our study combined with the crushed nature of *Pteranodon* remains from the Niobrara Formation limit our ability to analyze this further as few three dimensional specimens exist. Fastnacht [24] reported a lower range of ASP in dsungaripteroid pterosaurs, although it is unknown which bones or specimens his conclusions were based on. Dsungaripteroids have thick-walled bones compared to other pterosaurs [27], which explains the lower value of ASP [24]. This may be relevant to the debated taxonomic nature of *Bennettazhia,* the humerus studied here (USNM 11925). Although this specimen is typically referred to as an azhdarchid [28], the lack of proximal pneumatic foramina characteristic of azhdarchids suggests it may be better to consider it an indeterminate Tapejaroidea (Dsungaripteroidea + Azhdarchoidea) (M. Witton, pers. comm.). Alternatively, it may represent a different group, which is suggested by a recent phylogenetic analysis that does not support the traditional grouping of Tapejaroidea [29]. Another explanation for the difference in values may come from the method of converting *K* values to ASP in pterosaurs. While *K* ^2^ is equal to ASP for semi-circular shaft cross sections with constant wall thickness such as those seen in most birds, pterosaur wing phalanges are sub-triangular in cross section with thickness increasing towards the corners. As it is unclear which bones the dsungaripterid values came from [24], this difference could be from converting non-circular bone cross sections to ASP values which may not be accurate. Comparison with data published from SMNK PAL 3985 [17] demonstrated that the rib, femur, tibia, and cervical vertebra ASP ranges are similar to those found in this study. However, the only bone in SMNK PAL 3895 directly comparable here is a wing phalanx, and represents the smallest ASP found with only 0.27 (Table 3), notably different from wing phalanx ASP found here (Table 2). An ASP of 0.27 was determined by taking a picture of a cross section of the specimen which had the internal structure exposed, and applied the calculated ASP from this single cross section throughout the bone [17]. It may be that a more comprehensive study of this specimen using CT analysis would reveal a larger ASP more characteristic of other bones. Further analysis of this specimen is needed to determine its pneumatic properties in more detail.

When comparing the results here with those of studies estimated from *K* values of the long bones of birds, pterosaur ASP exhibits a narrower range than that of birds (Tables 2, 3). The pterosaur results are at the high end of the bird range, and the extreme values are greater than the highest bird ASP estimates. This adds further support to the view that pterosaurs were highly pneumatic, which may have been linked to their well developed respiratory system and possibly contributed to their ability to reach gigantic sizes [6].

In comparison with sauropod dinosaurs, the ASP of pterosaur wing bones is similar to that of the high end of the range seen in sauropod vertebrae (Tables 2, 3). As only wing elements were studied here, a direct comparison between comparable elements in sauropods and pterosaurs is not possible, and is a subject that holds promise for future study. In summary, it appears that the wing elements of pterodactyloid pterosaurs possess higher ASP values than the long bones of birds, and are comparable to the most pneumatized sauropod vertebrae.

### ASP Patterns in Pterosaur Wing Bones

Although not statistically significant due to the small number of samples, it appears that ASP decreases (and therefore the proportion of bone volume increases) distally along the phalanges (Table 2, Fig. 2). UP WP1 and NHMUK PV OR39411 show higher ASP values throughout the bone than that of UP WP2. NHMUK PV OR41637 has a similar distribution throughout the shaft of the bone to UP WP2, but has a higher ASP in the proximal head. However, UP WP3 has lower ASP than all other specimens, barely overlapping with any other specimens, while still sharing the general pattern of highest ASP at the ends. This suggests that the pterosaur wing phalanges are more highly pneumatized proximally, and decrease in pneumatisation distally. However, while UP WP2 and WP3 are from the same individual, UP WP1 is not. Further investigation is required to determine if pneumaticity does actually decrease distally, or if the results reported here represent individual variation.

### Implications of ASP Variation within Bones

Although comparison of degrees of pneumaticity within taxa is valuable, the more important finding of this study is the degree of variation of ASP within individual bones (Fig. 2). Previous studies have reported ASP or *K* values from a single cross section through the bone that is generally in the shaft, either from the specimen itself, an image, or in rare cases from a single CT scan slice [17], [20]–[24]. This study shows that ASP is not constant throughout a bone: ASP is higher in the heads than in the shafts. This was not expected as the heads of the bones are filled with spongy trabecular bone, but the very thin walls of the trabeculae mean that there is a large proportion of air space. Also, increased diameter in the heads is coupled with decreased cortical thickness towards the heads (as much as 50% decrease [30]).

The difference between the average ASP and the smallest ASP measured in each specimen is 5–19%, while the difference between the highest and lowest measurements is 10–29% (table 2). These differences highlight the problem of using a single section to quantify pneumaticity within a bone since the shaft consistently shows a lower ASP than the heads. Although this trend is clear for pterosaur wing phalanges, it is not in the humerus USNM 11925 (Table 2). It is unknown if this is a general feature of humeri, or this single taxon and more investigation is needed.

It is also unknown if variation of ASP throughout a bone is solely characteristic of pterosaurs, or if it is present in other animals with pneumaticity, such as birds and sauropods. Further work must therefore be done on both sauropods and birds to determine if the variation is present within these groups as well as pterosaurs, and to what extent ASP may have been underestimated in these taxa. As biomechanical studies are often based on shaft cross-sections, this has implications for the use of a shaft cross-section to determine the bone-to-air ratio. This result may also apply to apneumatic bones, those that are filled with marrow, which were not studied here, and require more study.

### Variation of Pneumaticity in Larger Flying Animals

Larger flying birds consistently have higher pneumaticity than smaller flying birds, and in groups with variable pneumaticity, the largest species always show higher pneumaticity [12], [19]. ASP is also higher in larger animals, as shown by large pterosaurs consistently having higher ASP than birds. This reveals a general trend in flying animals to increase ASP as they become larger. Traditionally, pneumaticity in birds has been attributed to reducing mass by hollowing out the bones, but this is likely to be only one of several possibly entangled factors. Another plausible driver is proposed here. Geometric considerations show that as ASP increases, specific stiffness (stiffness per unit mass) also increases. Young’s Modulus (material stiffness) of bone also increases with increasing density [31] implying that bones with higher density are also more stiff in bending. As bird and bat wing bones may be more dense than equivalent mammal bones (without taking into account the added density from marrow in apneumatic bones) [32], this suggests an evolutionary pressure to increase the stiffness of bones in flying animals. The stiffness of a bone is the result of the Young’s Modulus of the bone, and the shape of its cross-section, with bending stiffness increasing rapidly as the diameter of a bone increases. This leads to a strong new hypothesis: ASP increases with size in flying animals as an adaptation to increase bone bending stiffness related to the pressures of flight. The resulting decrease in bone mass may be a beneficial by-product caused by the decrease in bone volume and loss of marrow that come with pneumaticity.

## Conclusions

By using results obtained from the CT scanning of pterosaur wing bones, we have for the first time been able to quantify the variation of pneumaticity as a continuous variable over the full length of fossil bones. Significant variation of ASP within individual bones is revealed, suggesting that previous estimates of sauropod, bird, and pterosaur pneumaticity based on inspection of single bone cross sections may significantly underestimate the degree of pneumaticity. Our results demonstrate that this is true for pterosaur wing bones, but further research is required to confirm the result in sauropods, birds, and in the axial skeleton of pterosaurs. We further provide evidence that pterosaurs were more pneumatic than birds, which we propose is due to the increased pressures of flight in larger animals, specifically related to bending stiffness, while weight reduction is a secondary advantage.

## Supporting Information

Table S1 **Tables of ASP calculations in eight pterosaur wing bones.**
(XLSX)
